# FSH may be a useful tool to allow early diagnosis of Turner syndrome

**DOI:** 10.1186/s12902-018-0236-4

**Published:** 2018-02-07

**Authors:** Stela Carpini, Annelise Barreto Carvalho, Sofia Helena Valente de Lemos-Marini, Gil Guerra-Junior, Andréa Trevas Maciel-Guerra

**Affiliations:** 1 Department of Pediatrics, Faculty of Medical Sciences (FCM), State University of Campinas (Unicamp), São Paulo, Brazil; 2 Post-Graduate Program in Child and Adolescent Health, FCM, Unicamp, São Paulo, Brazil; 3 Department of Medical Genetics, FCM, Unicamp, Rua Tessalia Vieira de Camargo, 126, Campinas, SP 13083-887 Brazil

**Keywords:** Turner syndrome, Gonadal dysgenesis, Puberty delayed, Follicle-stimulating hormone, Luteinizing hormone

## Abstract

**Background:**

Ultrasensitive assays to measure pre-pubertal gonadotropins levels could help identify patients with Turner syndrome (TS) in mid-childhood, but studies in this field are scarce. The aim of this study was to analyze gonadotropins levels in girls with TS throughout childhood.

**Methods:**

Retrospective longitudinal study conducted with 15 girls with TS diagnosed with < 5 years whose FSH and LH measures were available since then. Hormones were evaluated in newborn/mini-puberty (< 0.5 years), early childhood (0.5–5 years), mid-childhood (5–10 years) and late childhood/adolescence (> 10 years). In newborn/mini-puberty and late childhood/adolescence pre-pubertal or pubertal gonadotropins were considered normal; in early childhood and mid-childhood concentrations above the pre-pubertal range were considered abnormal.

**Results:**

Abnormally high FSH alone was found in four of five patients in newborn/mini-puberty, 13 of 15 during early childhood and nine of 15 during mid-childhood. In the group of 12 patients in late childhood/adolescence, the three girls with spontaneous puberty had only normal levels; the remaining showed only post-menopausal concentrations. In mid-childhood one patient exhibited only pre-pubertal FSH. Conversely, most LH measurements in early and mid-childhood were normal.

**Conclusion:**

Karyotyping of girls with short stature and high FSH levels would allow early diagnosis of Turner syndrome in a significant number of patients, particularly when resources for chromosome study of all girls with growth deficiency are limited.

**Electronic supplementary material:**

The online version of this article (10.1186/s12902-018-0236-4) contains supplementary material, which is available to authorized users.

## Background

Even now, in the genomics era, some genetic disorders remain a challenge to diagnosis due to wide phenotypic variability and/or lack of widespread availability of genetic tests, particularly in developing countries. This is the case with Turner syndrome (TS), which has an incidence of 1:2,130 female newborns [1] and is characterized by the presence of a normal X chromosome and partial or total loss of the other sex chromosome, X or Y. Although traditionally associated with the 45,X karyotype, TS can also be due to mosaicism or structural abnormalities of sex chromosomes. There are also strong indications that patients with a 45,X karyotype are actually mosaics (cryptic mosaicism) [2].

The clinical picture varies widely, and includes dysmorphic features of face, neck, chest and limbs. Cardiovascular and renal/collecting system anomalies may also be found, as well as autoimmune thyroid disease. Nonetheless, the most constant features are short stature and primary hypogonadism due to gonadal dysgenesis [3].

Gonadal dysgenesis in TS is the result of massive apoptosis of the oocytes during fetal life [4–6]. Though the large majority of women with TS have dysgenetic gonads, about 30% will undergo some spontaneous pubertal development, and 2–5% may achieve spontaneous pregnancy [7, 8].

A study in the 1970’s revealed that plasma concentrations of follicle-stimulating hormone (FSH) and luteinizing hormone (LH) in TS patients show a biphasic pattern [9]. In that study, mean basal plasma FSH level was strikingly elevated from 2 days to 4 years; thereafter, a decline in plasma FSH to pre-pubertal levels occurred between 4 and 10 years, followed by a rise after 10 years, reaching postmenopausal levels some years later. The pattern of LH secretion was qualitatively similar to that of FSH, though the values for LH were 1/3 to 1/10 those for FSH. In that work, however, gonadotropins concentrations were determined by radioimmunoassay (RIA), which has low sensitivity.

The development of ultrasensitive immunochemiluminometric and immunofluorometric assays in the 1980’s and 1990’s demonstrated that pre-pubertal FSH and LH levels are much lower than previously thought [10]. The study of gonadotropins levels of girls with TS revealed that they remained high even at pre-pubertal age, and it was even suggested that measurement of gonadotropins could help identify prepubertal patients with primary gonadal failure [11].

However, a study of 68 girls with TS, 20 of them followed over a period of 2 years, revealed normal FSH and LH concentrations in 9 and 23.5% of patients between 0 and 5 years, respectively, and in 41 and 74% of patients between 5 and 10 years, respectively [12].

As most available data were based on transversal studies, a retrospective longitudinal one was carried out with 70 girls aged 0 to 16 years; the median ages at TS diagnosis were 5.2 years in the group of patients with a 45,X karyotype and 8.2 years in the group of patients with other chromosome constitutions. Most patients, both those with and without spontaneous pubertal development, had at least one FSH and LH value within the reference range during mid-childhood [13].

Divergences among studies about various aspects of TS phenotype are common [14]; they are due to the wide karyotypic variability of TS, including mosaicism, various structural abnormalities and different proportions of normal and abnormal cell lines. The same must apply to the extent of ovarian dysgenesis in these girls and hence to differences in the levels of gonadotropins in the pediatric age range. Thus, more data are needed to draw definite conclusions about this matter.

The aim of this work was to analyze FSH and LH levels in a sample of girls with TS who were diagnosed in early childhood (0–5 years) and followed thereafter.

## Methods

A retrospective longitudinal study was conducted with patients with TS who were diagnosed since the mid-1990’s with less than 5 years of age, and whose measures of FSH and LH levels were available both in early childhood and in the following years, with routine follow-up visits scheduled every 6 months. FSH and LH levels had been measured using electrochemiluminescence assays as part of their routine clinical follow-up since the time of diagnosis; these data were obtained retrospectively from patients’ files. In the absence of spontaneous puberty, only gonadotropins levels measured prior to sex hormone replacement therapy (HRT) were included.

FSH and LH levels were classified as pre-pubertal (< 3.8 mUI/mL and < 1.4 mUI/mL, respectively), post-menopausal (> 25.8 mUI/mL and > 7.7 mUI/mL, respectively) or pubertal (those within the intermediate range) according to reference values of the assays used in our service (Roche Elecsys®). Gonadotropins levels were evaluated according to age range: newborn/mini-puberty (0–0.5 years), early childhood (0.5–5 years), mid-childhood (5–10 years) and late childhood/adolescence (> 10 years). In newborn/minipuberty and in late childhood/adolescence FSH and LH levels were considered normal when they were within the prepubertal or pubertal range, while in early and mid-childhood any concentrations above the pre-pubertal ones were regarded as abnormal.

The SPSS for Windows software, version 20 (SPSS, Inc., Chicago, IL, USA) was used for data analysis. This study was carried out in accordance with the Declaration of Helsinki and the protocol was approved by the Research Ethics Committee of the State University of Campinas (931/2008; CAAE 0352.0.146.000–08). The study was exempt from written informed consent from the subjects due to its retrospective design and noninterventional nature of the study protocol.

## Results

Fifteen out of 186 patients diagnosed since the mid-1990’s (8%) met the inclusion criteria (Table 1). Three of them were followed only until mid-childhood and the remaining until adolescence. Mean age at diagnosis was 1.7 years, and mean age at last hormonal evaluation (that of the last visit – in prepubertal patients and those with spontaneous puberty – or the last measurement prior to initiation of HRT) was 11.4 years. The mean follow-up was 10.7 years (range: 3.0–16.5 years), and most patients had a 45,X karyotype.

**Table 1:** Description of the sample: age, karyotype, puberty and results of gonadotropins measurements

Patient	Age at diagnosis	Age at last hormone evaluation^a^	Karyotype	Pubertal status	Abnormal measurements/total number of measurements	
					FSH	LH
1	NB/MP	LC/A	45,X	Pre-pubertal	9/10	2/10
2	NB/MP	LC/A	45,X	Induced puberty	11/11	3/12
3	NB/MP	LC/A	45,X/46,X,+mar (*SRY* -)	Induced puberty	16/17	10/17
4	NB/MP	LC/A	45,X	Induced puberty	10/11	6/9
5	NB/MP	LC/A	45,X	Pre-pubertal	12/12	3/12
6	NB/MP	LC/A	45,X	Spontaneous puberty^b^	5/14	0/14
7	NB/MP	LC/A	45,X	Induced puberty	10/10	8/10
8	EC	LC/A	45,X/46,XX	Spontaneous puberty^b^	0/21	4/21
9	EC	MC	45,X	Pre-pubertal	3/3	2/3
10	EC	LC/A	45,X	Induced puberty	10/11	4/10
11	EC	LC/A	45,X	Pre-pubertal	6/6	1/6
12	EC	LC/A	45,X/46,XX	Spontaneous puberty	2/10	0/8
13	EC	MC	45,X	Pre-pubertal	2/2	1/2
14	EC	LC/A	45,X	Induced puberty	9/9	5/10
15	EC	MC	45,X/46,X,i(Xq)	Pre-pubertal	4/4	0/4

*EC* early childhood, *LC/A* late childhood/adolescence, *MC* midchildhood, *mo* month, *NB/MP* newborn/minipuberty, *yr.* year

^a^ That of the last visit – in prepubertal patients and those with spontaneous puberty – or the last measurement prior to initiation of hormone replacement therapy

^**b**^ Menarche already occurred

Three patients had spontaneous pubertal development, one of them with a 45,X chromosome constitution in 50 cells and two with mosaicism; two had already had menarche. Six had primary hypogonadism (absence of pubertal signs and elevated gonadotropins levels) and had already initiated HRT. Three were adolescents who already had post-menopausal FSH levels but were not under HRT (patients 1, 5 and 11); the remaining had not reached adolescence at the time this study was conducted, but in one of them a post-menopausal FSH level was already detected. The number of gonadotropins measurements per patient ranged from two to 21 (median: 10) (Table 1).

**Fig. 1:**
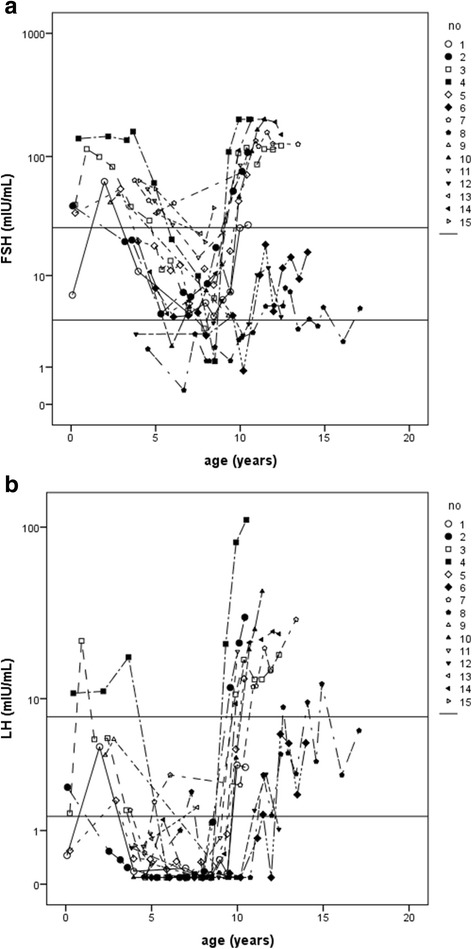
Gonadotropins levels in different ages in girls with Turner syndrome. Hormone values are presented in the y-axis on a logarithmic scale. The lines on the y-axis represent the lower and upper normal limits for **a)** FSH and **b)** LH concentrations. Levels above the upper limits were considered post-menopausal and those below the lower limits were regarded as pre-pubertal

Regarding FSH, four of the five patients whose gonadotropins were measured in the first 6 months of life had abnormally high levels; none of the five girls developed spontaneous puberty. During early childhood, 13 out of 15 girls had no measurements in the normal pre-pubertal range; moreover, nine of these 13 patients had at least one measurement which was in the post-menopausal range. During mid-childhood, six of the 15 girls had at least one normal FSH concentration, but three of them also exhibited at least one post-menopausal concentration in this period; a single patient [8] exhibited only prepubertal FSH levels. Ninety-three percent of the measurements performed in early childhood and 80% of those performed in mid-childhood were abnormal. In the group of 12 patients > 10 years old, those without spontaneous pubertal development showed only post-menopausal concentrations (Fig. 1, Table 2 and Additional file 1).

**Table 2:** Results of gonadotropins measurements according to age range in TS girls

				measurements						
Age range (years)	N	mean	range	PP	P	PM	Total	Patients with at least one normal level for age^a^	Patients with only normal levels for age^a^	Patients with at least one abnormal level for age^a^
FSH										
0–0.5	5	52.05 ±51.03	6.65–140.00	0	1	4	5	1	1	4/5 (80%)
0.5–5	15	52.09 ±42.35	1.80–160.00	2	8	20	30	2	2^b^	13/15 (87%)
5–10	15	18.72 ±32.87	0.30–201.00	13	42	11	66	6	1^b^	14/15 (93%)
> 10	12	65.16 ±69.55	0.87–201.00	8	18	24	50	3	3^b^	9/12 (75%)
LH										
0–0.5	5	3.16 ±4.35	0.45–10.80	2	2	1	5	4	2	3/5 (60%)
0.5–5	15	3.07 ±5.37	0.09–22.20	18	8	3	29	13	9	6/15 (40%)
5–10	15	2.60 ±10.58	0.09–82.02	53	7	5	65	13	5	10/15 (67%)
> 10	12	12.97 ±17.36	0.09–110.00	7	17	25	49	5	3	9/12 (75%)

*FSH* follicle-stimulating hormone, *LH* luteinizing hormone, *N* number of patients, *P* pubertal, *PM* post-menopausal, *PP* pre-pubertal

^a^ 0–0.5 years: PP or P level; 0.5–5 years and 5–10 years: PP level; > 10 years: PP or P level

^b^ only patients with spontaneous pubertal development

Only one out of five girls had an abnormally high LH concentration detected in the first 6 months of life, and most had at least one normal measurement in early childhood and mid-childhood. Sixty-two percent of the measurements performed in early childhood and 82% of those performed in mid-childhood were in the normal pre-pubertal range. In late childhood/adolescence, five patients had at least one pre-pubertal or pubertal level, though two of them also had at least one post-menopausal concentration, including one of the girls with spontaneous pubertal development (patient 8); all the remaining showed only post-menopausal concentrations (Fig. 1, Table 2 and Additional file 1).

FSH levels in this sample expressed the biphasic age pattern: they were high in newborn/mini-puberty and early childhood, declined in mid-childhood, though rarely reaching the normal pre-pubertal range, and then increased again in late childhood/adolescence. In comparison, the pattern of LH, though also biphasic, revealed much higher levels in late childhood/adolescence than in newborn/mini-puberty and early childhood. In addition, in mid-childhood the decline of LH compared to the previous period was less striking than that of FSH, and pre-pubertal levels were often seen (Fig. 1 and Table 2).

## Discussion

Gonadotropins levels in this sample expressed the same biphasic age pattern found by Conte et al. [9]; however, the use of an ultrasensitive assay in the present study revealed that, different from what was observed by those authors, the decline in plasma FSH seldom reaches pre-pubertal levels in early and mid-childhood. The significantly high mean FSH levels found in pre-pubertal TS girls in this sample were similar to those found by others [12]. Thus, our results indicate that many girls with TS could be diagnosed earlier if FSH measurements were routinely done in girls with unexplained short stature.

The results of our study differ from the other retrospective longitudinal study found in literature [13], in which most patients had at least one FSH and LH value within the reference range during mid-childhood. However, comparison between these studies is difficult. In fact, age at diagnosis varied widely in that study (44 of the 70 girls were more than 12 years old at diagnosis) and longitudinal samples could not be obtained in all cases; in addition, length of follow-up was also variable.

A survey conducted in our university hospital in the beginning of the 1990’s revealed that seven out of 38 girls with growth deficiency (18%) had TS [15]. Another study on this matter conducted with 353 subjects in China in the same decade found a very similar figure: 19% of the females had TS [16]. However, in other studies this proportion varied widely, from 4.5 to 81.7% [17–19].

Some authors have recommended routine karyotype analysis for all of these girls [20, 21], while others suggest that routine cytogenetic analysis should be restricted to those with associated congenital anomalies [19, 22], but this is still a matter of debate. In any case, karyotyping is not always available; this is particularly true in developing countries, in which the karyotype is an expensive exam and rarely offered in public health services. In addition, TS has wide phenotypic variability, and some features may not be recognized unless specifically sought [23]. As a consequence, TS may be diagnosed only in adolescence, when lack of pubertal development manifests in addition to short stature. In our institution, for instance, a recent survey revealed that mean age at diagnosis is 12 years, due to late referral by primary health services [24].

It is currently known that up to 40% of TS girls may have some degree of spontaneous pubertal development and up to 19% may have complete puberty and menses [7]; these figures may be even higher when the sample is not biased towards patients with hypogonadism [25]. Thus, some patients with residual ovarian function may have normal gonadotropins levels throughout childhood and escape diagnosis when these hormones are measured.

The majority, however, would benefit from the inclusion of FSH as an additional diagnostic tool to assess girls with unexplained growth deficiency. It has already been proposed that elevated FSH levels in childhood should prompt cytogenetic evaluation [21]. Indeed, in our sample normal measurements were uncommon both in early childhood and in mid-childhood; in addition, normal levels after 0.5 years were restricted to patients with spontaneous pubertal development. On the other hand, as most LH measurements in these age ranges were normal, its usefulness is limited.

Even though FSH measurement is not always sensitive for the diagnosis of TS in girls with unexplained short stature, this widely available and highly specific test to detect gonadal dysgenesis may lead to early diagnosis and prompt treatment in a significant number of patients by giving priority to performing their karyotype when this test is not accessible to screen all girls with growth deficiency. Even though gonadal dysgenesis is also a feature of other disorders of sex development, these conditions are usually not associated with short stature. This is the case of complete gonadal dysgenesis, either 46,XY (which may be due to mutations in *SRY* gene, among others) or 46,XX (which may be due to mutations in FSH receptor gene, among others). Thus, in some cases a normal karyotype may be found and lead to further investigations on the origin of high FSH levels.

Some of the strengths of this study are early age of diagnosis (less than 5 years in all cases), which avoids bias caused by pubertal delay as the main feature leading to clinical suspicion, and significantly long-term follow-up conducted in the same service. On the other hand, sample size is small due to definition of early age at diagnosis as an inclusion criterion.

## Conclusion

In summary, follow-up of 15 girls with TS from early infancy on revealed that most FSH measurements were above the normal range for age before late childhood/adolescence. These results indicate that inclusion of this test in the guidelines for the evaluation of girls with unexplained short stature would allow early diagnosis and treatment in a significant number of patients by prioritizing their cytogenetic evaluation, particularly when resources for chromosomal studies of all girls with growth deficiency are limited.

## Additional file

Additional file 1: Results of gonadotropins measurements according to age in TS girls. (DOCX 20 kb)

## Abbreviations

FSH: Follicular stimulating hormone
HRT: Sex hormone replacement therapy
LH: Luteinizing hormone
TS: Turner syndrome
