# Supplementary material for: FSH may be a useful tool to allow early diagnosis of Turner syndrome
Source: BMC Endocr Disord. 2018 Feb 7;18:8. doi: 10.1186/s12902-018-0236-4 (PMC5803864; doi:10.1186/s12902-018-0236-4)
Supplement: Additional file 1: — Results of gonadotropins measurements according to age in TS girls. (DOCX 20 kb) [file 12902_2018_236_MOESM1_ESM.docx]

| **patient** | **Age**  **(years)** | **FSH (mIU/mL)** | **LH**  **(mIU/mL)** |
| --- | --- | --- | --- |
| 1 | 0.08 | 6.65 | 0.45 |
| 1 | 1.97 | 62.30 | 4.90 |
| 1 | 3.97 | 10.87 | 0.18 |
| 1 | 6.98 | 4.40 | 0.23 |
| 1 | 7.95 | 5.62 | 0.09 |
| 1 | 8.45 | 4.16 | 0.09 |
| 1 | 8.96 | 6.01 | 0.37 |
| 1 | 9.42 | 7.12 | 0.15 |
| 1 | 9.98 | 25.67 | 3.66 |
| 1 | 10.46 | 27.25 | 3.53 |
| 2 | 0.10 | 39.20 | 2.50 |
| 2 | 2.52 | NA | 0.53 |
| 2 | 3.18 | 19.71 | 0.37 |
| 2 | 3.59 | 20.30 | 0.24 |
| 2 | 5.31 | 4.39 | 0.09 |
| 2 | 6.63 | 7.04 | 0.09 |
| 2 | 7.07 | 6.40 | 0.09 |
| 2 | 8.05 | 8.46 | 0.18 |
| 2 | 8.57 | 17.53 | 1.23 |
| 2 | 9.59 | 52.00 | 11.70 |
| 2 | 10.11 | 75.60 | 21.60 |
| 2 | 10.43 | 108.00 | 30.50 |
| 3 | 0.24 | 40.10 | 1.50 |
| 3 | 0.92 | 115.00 | 22.20 |
| 3 | 1.67 | 99.30 | 5.50 |
| 3 | 2.43 | 82.20 | 5.60 |
| 3 | 3.53 | 38.60 | 1.60 |
| 3 | 4.63 | 29.60 | 0.09 |
| 3 | 5.36 | 11.27 | 0.09 |
| 3 | 5.88 | 13.50 | 0.09 |
| 3 | 6.92 | 4.72 | 0.10 |
| 3 | 7.87 | 3.10 | 0.09 |
| 3 | 8.45 | 6.22 | 0.13 |
| 3 | 9.89 | 106.50 | 10.62 |
| 3 | 10.38 | 118.00 | 17.20 |
| 3 | 11 | 86.10 | 13.10 |
| 3 | 11.42 | 115.00 | 13.11 |
| 3 | 11.94 | 114.00 | 15.00 |
| 3 | 12.42 | 122.60 | 18.38 |
| 4 | 0.44 | 140.00 | 10.80 |
| 4 | 2.19 | 146.00 | 11.10 |
| 4 | 3.29 | 136.20 | NA |
| 4 | 3.66 | 160.00 | 17.80 |
| 4 | 4.91 | 60.60 | NA |
| 4 | 5.95 | 20.60 | 0.09 |
| 4 | 7.50 | 9.93 | 0.14 |
| 4 | 8.50 | 1.23 | 0.09 |
| 4 | 9.32 | 109 | 21.30 |
| 4 | 9.92 | 201.00 | 82.02 |
| 4 | 10.53 | 201.00 | 110.00 |
| 5 | 0.24 | 34.30 | 0.54 |
| 5 | 2.92 | 54.20 | 1.95 |
| 5 | 3.96 | 19.83 | 0.39 |
| 5 | 4.92 | 18.12 | 0.38 |
| 5 | 5.88 | 11.07 | 0.21 |
| 5 | 6.45 | 12.38 | 0.34 |
| 5 | 6.99 | 5.54 | 0.09 |
| 5 | 7.89 | 11.48 | 0.33 |
| 5 | 8.43 | 8.24 | 0.13 |
| 5 | 9.40 | 16.35 | 0.91 |
| 5 | 9.90 | 43.03 | 4.72 |
| 5 | 10.39 | 70.76 | 13.23 |
| 5 | 10.89 | 122.90 | 28.61 |
| 5 | 11.39 | 141.80 | 36.17 |
| 6 | 4.99 | 7.68 | 0.09 |
| 6 | 6.06 | 4.10 | 0.09 |
| 6 | 6.93 | 4.22 | 0.09 |
| 6 | 7.49 | 4.51 | 0.09 |
| 6 | 7.99 | 2.60 | 0.09 |
| 6 | 9.56 | 4.21 | 0.15 |
| 6 | 10.19 | 0.87 | 0.09 |
| 6 | 11.17 | 10.09 | 0.81 |
| 6 | 11.50 | 18.59 | 1.46 |
| 6 | 11.97 | 4.64 | 0.09 |
| 6 | 12.50 | 11.66 | 5.95 |
| 6 | 12.99 | 14.49 | 5.17 |
| 6 | 13.49 | 9.32 | 2.18 |
| 6 | 13.98 | 15.99 | 5.21 |
| 7 | 3.75 | 63.80 | 1.60 |
| 7 | 4.59 | 43.60 | 0.50 |
| 7 | 5.17 | 35.40 | 1.90 |
| 7 | 6.08 | 41.40 | 3.10 |
| 7 | 10.17 | 74.90 | 2.60 |
| 7 | 10.92 | 134.00 | 11.82 |
| 7 | 11.09 | 121.00 | 11.95 |
| 7 | 11.59 | 157.00 | 20.10 |
| 7 | 11.92 | 127.00 | 14.73 |
| 7 | 13.42 | 126.00 | 29.60 |
| 8 | 4.54 | 1.80 | 0.10 |
| 8 | 6.67 | 0.30 | 1.00 |
| 8 | 7.33 | 2.70 | 2.30 |
| 8 | 8 | 1.24 | 0.09 |
| 8 | 8.17 | 1.24 | 0.09 |
| 8 | 8.50 | 1.90 | 0.09 |
| 8 | 9.42 | 1.25 | 0.09 |
| 8 | 9.92 | 2.32 | 0.09 |
| 8 | 10.09 | 2.47 | 0.09 |
| 8 | 10.75 | 2.80 | 0.09 |
| 8 | 11.50 | 5.20 | 3.10 |
| 8 | 12 | 5.30 | 1.43 |
| 8 | 12.53 | 5.24 | 4.36 |
| 8 | 12.67 | 7.75 | 8.86 |
| 8 | 12.95 | 7.14 | 4.44 |
| 8 | 13.42 | 3.06 | 3.16 |
| 8 | 14.09 | 3.89 | 9.50 |
| 8 | 14.59 | 3.29 | 3.88 |
| 8 | 14.92 | 5.08 | 12.31 |
| 8 | 16.08 | 2.22 | 3.10 |
| 8 | 17.09 | 4.97 | 6.28 |
| 9 | 2.31 | 41.80 | 4.30 |
| 9 | 2.80 | 48.90 | 5.50 |
| 9 | 9.30 | 4.20 | 0.09 |
| 10 | 3.95 | 19.63 | 0.09 |
| 10 | 5.95 | 1.95 | 0.09 |
| 10 | 7 | 5.16 | 0.09 |
| 10 | 7.49 | 4.66 | NA |
| 10 | 7.98 | 7.30 | 0.10 |
| 10 | 8.91 | 12.50 | 0.16 |
| 10 | 9.41 | 7.08 | 0.09 |
| 10 | 9.93 | 46.60 | 4.12 |
| 10 | 10.69 | 110.00 | 19.71 |
| 10 | 11.01 | 165.00 | 25.90 |
| 10 | 11.45 | 200.00 | 43.00 |
| 11 | 4.52 | 54.30 | 0.63 |
| 11 | 4.93 | 43.37 | 0.92 |
| 11 | 6.01 | 27.36 | 0.12 |
| 11 | 7.53 | 14.41 | 0.09 |
| 11 | 8.99 | 23.66 | 0.82 |
| 11 | 10.01 | 84.84 | 19.21 |
| 11 | 13.08 | 103.20 | 32.51 |
| 12 | 3.83 | 2.70 | 0.60 |
| 12 | 7.92 | 2.71 | 0.09 |
| 12 | 8.42 | 3.56 | NA |
| 12 | 8.91 | 5.90 | 0.09 |
| 12 | 9.58 | 4.20 | 0.09 |
| 12 | 10.17 | 2.61 | 0.09 |
| 12 | 10.50 | 3.47 | NA |
| 12 | 11 | 10.16 | 1.59 |
| 12 | 11.67 | 11.70 | 3.14 |
| 12 | 12.42 | 4.10 | 1.03 |
| 13 | 4.99 | 34.00 | 0.80 |
| 13 | 7.67 | 23.10 | 1.70 |
| 14 | 4.67 | 10.80 | 0.09 |
| 14 | 5.68 | 4.40 | 1.30 |
| 14 | 6.96 | 5.90 | 0.09 |
| 14 | 8.54 | 10.44 | 0.09 |
| 14 | 9.25 | NA | 0.48 |
| 14 | 9.92 | 112.00 | 9.30 |
| 14 | 10.69 | 201.00 | 21.70 |
| 14 | 11.41 | 201.00 | 22.60 |
| 14 | 12.07 | 191.00 | 25.22 |
| 14 | 12.43 | 151.00 | 24.38 |
| 15 | 4.04 | 63.28 | 0.65 |
| 15 | 4.99 | 54.24 | 0.30 |
| 15 | 7.93 | 19.52 | 0.09 |
| 15 | 8.44 | 37.76 | 1.19 |
| 15 | 9 | 37.26 | 1.70 |
| 15 | 9.50 | 50.99 | 7.83 |
| 15 | 10 | 20.00 | 1.88 |

FSH= follicle-stimulating hormone; LH= luteinizing hormone; NA= not available
